# The TSC Complex-mTORC1 Axis: From Lysosomes to Stress Granules and Back

**DOI:** 10.3389/fcell.2021.751892

**Published:** 2021-10-29

**Authors:** Ulrike Rehbein, Mirja Tamara Prentzell, Marti Cadena Sandoval, Alexander Martin Heberle, Elizabeth P. Henske, Christiane A. Opitz, Kathrin Thedieck

**Affiliations:** ^1^Laboratory for Metabolic Signaling, Institute of Biochemistry, Center for Molecular Biosciences Innsbruck, University of Innsbruck, Innsbruck, Austria; ^2^Brain Cancer Metabolism Group, German Consortium of Translational Cancer Research (DKTK) & German Cancer Research Center (DKFZ), Heidelberg, Germany; ^3^Faculty of Bioscience, Heidelberg University, Heidelberg, Germany; ^4^Section Systems Medicine of Metabolism and Signaling, Department of Pediatrics, University of Groningen and University Medical Center Groningen, Groningen, Netherlands; ^5^Pulmonary and Critical Care Medicine, Department of Medicine, Brigham and Women’s Hospital, Harvard Medical School, Boston, MA, United States; ^6^Department of Neurology, National Center for Tumor Diseases, University Hospital Heidelberg, Heidelberg, Germany; ^7^Department for Neuroscience, School of Medicine and Health Sciences, Carl von Ossietzky University Oldenburg, Oldenburg, Germany

**Keywords:** TSC complex, mTORC1 (mechanistic target of rapamycin complex 1), HDLBP, lysosomes, stress granules (SG), autophagy, lymphangioleiomyomatosis (LAM), G3BP1 (G3BP stress granule assembly factor 1)

## Abstract

The tuberous sclerosis protein complex (TSC complex) is a key integrator of metabolic signals and cellular stress. In response to nutrient shortage and stresses, the TSC complex inhibits the mechanistic target of rapamycin complex 1 (mTORC1) at the lysosomes. mTORC1 is also inhibited by stress granules (SGs), RNA-protein assemblies that dissociate mTORC1. The mechanisms of lysosome and SG recruitment of mTORC1 are well studied. In contrast, molecular details on lysosomal recruitment of the TSC complex have emerged only recently. The TSC complex subunit 1 (TSC1) binds lysosomes *via* phosphatidylinositol-3,5-bisphosphate [PI(3,5)P2]. The SG assembly factors 1 and 2 (G3BP1/2) have an unexpected lysosomal function in recruiting TSC2 when SGs are absent. In addition, high density lipoprotein binding protein (HDLBP, also named Vigilin) recruits TSC2 to SGs under stress. In this mini-review, we integrate the molecular mechanisms of lysosome and SG recruitment of the TSC complex. We discuss their interplay in the context of cell proliferation and migration in cancer and in the clinical manifestations of tuberous sclerosis complex disease (TSC) and lymphangioleiomyomatosis (LAM).

## Introduction

For cellular growth and survival, cells have to tightly balance their metabolism to adapt to nutritional changes and environmental stressors. The TSC complex (tuberous sclerosis protein complex) constitutes a key integrator of nutrient and stress signals (Huang and Manning, 2008; Demetriades et al., 2016; Liu and Sabatini, 2020), which adapts cellular metabolism to environmental conditions by suppressing the anabolic master regulator mTORC1 (mechanistic target of rapamycin complex 1; Mossmann et al., 2018; Tee, 2018; Kim and Guan, 2019; Hoxhaj and Manning, 2020; Liu and Sabatini, 2020). mTORC1 is an evolutionary highly conserved multi-protein complex. Apart from the MTOR kinase itself, mTORC1 contains the complex-specific interaction partners RPTOR (regulatory associated protein of MTOR complex 1) and AKT1S1 (AKT1 substrate 1) (Yip et al., 2010; Yang et al., 2013; Chao and Avruch, 2019). The TSC complex-mTORC1 axis translates nutrient and stress signals into tightly orchestrated cellular responses that impinge on anabolic processes including translation, as well as catabolic processes such as autophagy (Liu and Sabatini, 2020). Disturbances of the TSC complex lead to mTORC1 hyperactivation and have been linked to diseases including cancer and the clinical manifestations of tuberous sclerosis complex disease (TSC), which are both characterized by cellular overgrowth and aberrant migration (Orlova and Crino, 2010; Borkowska et al., 2011; Curatolo et al., 2015; Henske et al., 2016; Condon and Sabatini, 2019; Jozwiak et al., 2019). Lysosomes are widely recognized as the major signaling platform at which the TSC complex inhibits mTORC1. Also other inhibitory cues such as the RRAG GTPases (Ras related GTP binding proteins) and AMPK (AMP-activated protein kinase) suppress mTORC1 at lysosomes [reviewed in detail by Oakhill et al. (2010); Kim and Guan (2019); Gonzalez et al. (2020); Liu and Sabatini (2020); Fernandes and Demetriades (2021)]. A growing body of evidence shows that stress granules (SGs) constitute a non-membranous compartment at which mTORC1 is inhibited under stress through several mechanisms (Takahara and Maeda, 2012; Thedieck et al., 2013; Wippich et al., 2013; Ramiscal et al., 2015; Lastres-Becker et al., 2016; Pla-Martin et al., 2020; Mediani et al., 2021). Whereas the molecular machinery mediating the recruitment and regulation of mTORC1 at lysosomes (Rabanal-Ruiz and Korolchuk, 2018; Condon and Sabatini, 2019; Kim and Guan, 2019) or SGs (Takahara and Maeda, 2012; Thedieck et al., 2013; Wippich et al., 2013; Mediani et al., 2021) has been investigated in much detail, recent studies shed light on the mechanisms tethering the TSC complex to lysosomes (Fitzian et al., 2021; Prentzell et al., 2021) and to SGs (Kosmas et al., 2021). In this mini-review we summarize the latest findings focusing on the interplay of the TSC complex with SGs and lysosomes. We discuss the impact of this crosstalk in the context of TSC, lymphangioleiomyomatosis (LAM) and cancer.

## Main Text

### The Lysosomal TSC Complex and SGs Inhibit mTORC1

The TSC multiprotein complex consists of TSC complex subunit 1 (TSC1), TSC2, and TBC1 domain family member 7 (TBC1D7) (Dibble et al., 2012). The three subunits assemble with a 2:2:1 stoichiometry (Dibble et al., 2012; Ramlaul et al., 2021; Yang et al., 2021). The coiled-coil domains of two TSC1 proteins intertwine in parallel in a double-helix bundle that interacts *via* several sites with the TSC2 dimer (Ramlaul et al., 2021; Yang et al., 2021). The two TSC2 molecules interact *via* their dimerization domains in an antiparallel manner, allowing the catalytic pockets of the GAP (GTPase-activating protein) domains to face outward of the TSC complex (Yang et al., 2021). This asymmetric TSC1-TSC2 complex binds a single TBC1D7 molecule *via* association with one C-terminus in the TSC1 dimer.

**FIGURE 1:**
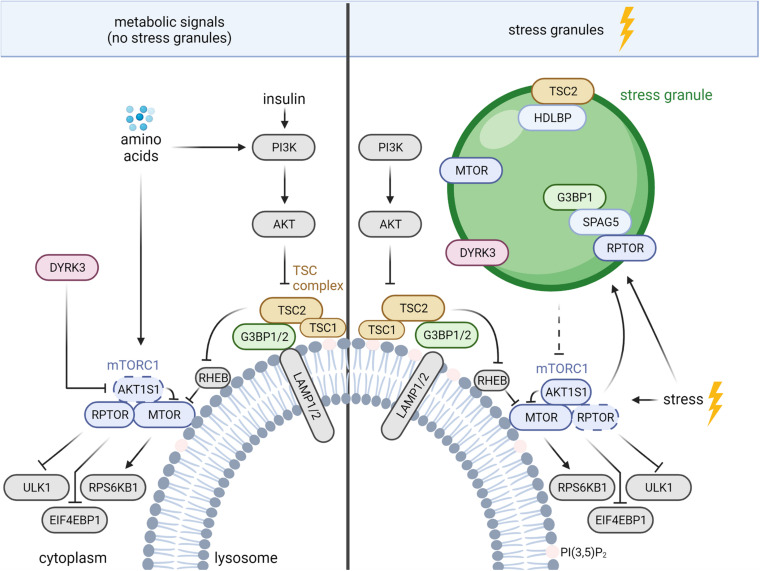
TSC complex-mTORC1 signaling during nutrient sufficiency and stress granule (SG) formation. The mechanisms are described in the text. AKT, AKT serine/threonine kinase; AKT1S1, AKT substrate 1; DYRK, dual specificity tyrosine phosphorylation regulated kinase 3; EIF4EBP1, eukaryotic translation initiation factor 4E binding protein 1; G3BP 1/2, stress granule assembly factor 1/2; HDLBP, high density lipoprotein binding protein; LAMP1/2, lysosomal associated membrane proteins 1/2; MTOR, mechanistic target of rapamycin kinase; PI3K, phosphoinositide 3-kinases; PI(3,5)P_2_, phosphatidylinositol-3,5-bisphosphate; RHEB, RAS homolog-mTORC1 binding; RPS6KB1, ribosomal protein S6 kinase B1; RPTOR, regulatory associated protein of MTOR complex 1; SPAG5, sperm associated antigen 5; TSC1/2, TSC complex subunit 1/2; ULK1, unc-51 like autophagy activating kinase 1. Dashed arrow, inhibition *via* disassembly of mTORC1.

In healthy cells, the TSC complex integrates signals from multiple growth factor pathways (Huang and Manning, 2008), as well as nutrient sufficiency and cellular stresses (Demetriades et al., 2014, 2016; Menon et al., 2014; Plescher et al., 2015; Carroll et al., 2016). In response to growth factors, including insulin, the AKT serine/threonine kinase (AKT) phosphorylates TSC2 and inhibits the TSC complex (Inoki et al., 2002; Manning et al., 2002; Potter et al., 2002). Apart from the activation of mTORC1, insulin-AKT signaling enhances a TSC2-independent function of TSC1 in cytostatic and pro-metastatic TGFB (transforming growth factor beta)-Smad2/3 (SMAD family member 2/3) signaling (Thien et al., 2015). Like AKT, also WNT (Wnt family member) (Inoki et al., 2006) and MAPK (mitogen-activated protein kinase) (Ma et al., 2005) signaling suppress TSC2 *via* RPS6KA1 (ribosomal protein S6 kinase A1) (Roux et al., 2004) and GSK3B (glycogen synthase kinase 3 beta), respectively. In contrast, phosphorylation of TSC2 by AMPK activates the TSC complex and inhibits mTORC1 (Inoki et al., 2003b). When growth factor signals are low, the TSC complex translocates to the lysosomal surface (Menon et al., 2014). Similarly, deprivation of all amino acids (Demetriades et al., 2014) or of arginine alone (Carroll et al., 2016) as well as hyperosmotic stress, hypoxia, pH stress and 2-Deoxy-D-glucose (Plescher et al., 2015; Demetriades et al., 2016) enhance the lysosomal association of the TSC complex. The TSC complex acts as a GAP that inhibits the small GTPase RHEB (RAS homolog-mTORC1 binding) by enhancing the conversion of RHEB‘s GTP-bound state to the GDP-bound state (Inoki et al., 2003a; Tee et al., 2003; Zhang et al., 2003). GTP-bound RHEB activates mTORC1, and the TSC complex suppresses mTORC1 upon growth factor shortage, nutrient deprivation, and other stresses (Huang and Manning, 2008; Plescher et al., 2015; Demetriades et al., 2016; Fernandes and Demetriades, 2021). Conversely, growth factor and nutrient sufficiency reduce the amount of lysosomal TSC complex (Demetriades et al., 2014; Menon et al., 2014; Carroll et al., 2016), increasing the abundance of RHEB-GTP and activating mTORC1 (Zhang et al., 2003; Menon et al., 2014). MTOR directly binds to RHEB-GTP, which causes a conformational change in the active site of the MTOR kinase domain (Yang et al., 2017). This allows mTORC1 to bind and phosphorylate its multiple substrates (Liu and Sabatini, 2020). Among them are EIF4EBP1 (eukaryotic translation initiation factor 4E binding protein 1) and RPS6KB1 (ribosomal protein S6 kinase B1), whose phosphorylation by mTORC1 enhances cap-dependent translation, and ULK1 (unc-51 like autophagy activating kinase 1) *via* which mTORC1 suppresses autophagy (Liu and Sabatini, 2020; Figure 1).

Beyond the lysosomal TSC complex, mTORC1 inhibition under stress is also mediated by SGs, cytoplasmic protein-RNA assemblies formed upon stress-induced inhibition of translation (Takahara and Maeda, 2012; Thedieck et al., 2013; Wippich et al., 2013; Heberle et al., 2015; Mediani et al., 2021). SGs constitute a dynamic non-membranous compartment that sorts mRNAs for maintenance or decay (Advani and Ivanov, 2019), controls signaling networks (Kedersha et al., 2013; Heberle et al., 2015), and promotes survival under stress (Kim et al., 2005; Arimoto et al., 2008; Tsai and Wei, 2010; Thedieck et al., 2013; Park et al., 2020). A variety of stress signals promote SG assembly *via* mechanisms associated with stalled translation [reviewed in detail by Alberti and Dormann (2019); Hofmann et al. (2021)]. The best described regulators of SG assembly are the eukaryotic translation initiation factor 2 subunit alpha (EIF2S1) kinases (Anderson et al., 2015), which inhibit EIF2S1 to diminish global cap-dependent translation (Holcik, 2015). The release of monosomal mRNAs enables the recruitment of RNA-binding proteins, such as the G3BP stress granule assembly factors 1 and 2 (G3BP1/2 or G3BPs), leading to SG assembly (Anderson et al., 2015; Panas et al., 2016). Pbp1, the yeast ortholog of Ataxin-2, acts under stress to recruit yeast RPTOR (Kog1) and MTOR (Tor1) to SGs (Takahara and Maeda, 2012). SGs also sequester MTOR in mammalian cells, but the recruiting protein remains unknown (Wippich et al., 2013; Figure 1). mTORC1 inhibition by SGs in mammalian cells is mediated by the sperm associated antigen 5 (SPAG5, also known as astrin) that recruits the mTORC1 specific scaffold protein RPTOR to SGs, and disassembles mTORC1 (Thedieck et al., 2013). In addition, SGs regulate mTORC1 *via* the dual specificity tyrosine phosphorylation regulated kinase 3 (DYRK3) (Wippich et al., 2013; Mediani et al., 2021; Figure 1). Under non-stressed conditions, cytosolic DYRK3 phosphorylates and represses mTORC1’s inhibitory subunit AKT1S1, leading to mTORC1 activation (Wippich et al., 2013). In response to stress SGs recruit inactive DYRK3, allowing active AKT1S1 to suppress mTORC1 (Wippich et al., 2013; Mediani et al., 2021). DYRK3 stabilizes SGs, enhancing inhibitory effects of SGs on mTORC1. Next to the inhibitory cues, activating stress inputs (Wang and Proud, 1997; White et al., 2007; Wu et al., 2011; Sfakianos et al., 2018; Heberle et al., 2019) finely orchestrate mTORC1 activity. mTORC1 enhances SG formation by several mechanisms that involve mediators of translation and autophagy (Fournier et al., 2013; Mazan-Mamczarz et al., 2015; Sfakianos et al., 2018; Zhang et al., 2018). *Via* such SG-mediated negative feedback mTORC1 may restrict its own activity under stress.

### Crosstalk of Lysosomes and SGs in TSC Complex-mTORC1 Signaling

Several findings indicate crosstalk between lysosomes and SGs. Absence of SPAG5 not only reduces SG tethering of RPTOR but also enhances its binding to lysosomes (Thedieck et al., 2013). In agreement, in the absence of SGs, the core SG proteins and *bona fide* markers of SG assembly G3BP1 and 2 (Riggs et al., 2020) reside at the cytoplasmic surface of lysosomes and function as tethers of the TSC complex (Prentzell et al., 2021; Figure 1). The C-terminal RGG (arginine–glycine–glycine) domain of G3BP1 binds to TSC2 and the N-terminal NTF2L domain of G3BP1 binds to the lysosomal associated membrane proteins 1/2 (LAMP1/2), bridging the TSC complex to the lysosomal surface (Prentzell et al., 2021). G3BPs suppress mTORC1 signaling in the presence as well as in the absence of nutrients (growth factors and amino acids). In keeping with a function in lysosomal tethering of the TSC complex, G3BP1 inhibition is sufficient to phenocopy loss of TSC2 with regard to (i) mTORC1 hyperactivity, (ii) increased cell size, and (iii) enhanced lysosomal MTOR localization (Prentzell et al., 2021). Next to G3BPs, also the RHEB and RRAG GTPases contribute to the lysosomal recruitment of the TSC complex (Demetriades et al., 2014; Menon et al., 2014; Carroll et al., 2016; Yang et al., 2020). G3BP1 and RHEB deficiency reduce lysosomal TSC2 localization to a similar extent, without additive effects (Prentzell et al., 2021), indicating that both mechanisms are required for efficient lysosomal recruitment of the TSC complex. Of note, G3BP1 deficiency does not activate mTORC1 signaling in the presence of SGs (Prentzell et al., 2021) suggesting that the G3BPs’ functions at lysosomes and in SGs are mutually exclusive. It is tempting to speculate that in response to stress G3BP proteins shuttle from the lysosomes to SGs. Thus, stress may reduce lysosomal tethering of the TSC complex by G3BP to sustain mTORC1 activity. However, the TSC complex suppresses mTORC1 also under stress (Plescher et al., 2015; Demetriades et al., 2016) and mechanisms other than G3BP-TSC2 may take over for the lysosomal tethering of the TSC complex. Findings of Fitzian et al. (2021) suggest the involvement of lysosomal phospholipids as TSC1 binds PI(3,5)P2 (phosphatidylinositol-3,5-bisphosphate) in a charge dependent manner (Figure 1). Osmotic stress enhances PI(3,5)P2 levels in the lysosomal membrane (Jin et al., 2017), and it is conceivable that lysosomal tethering of the TSC complex *via* TSC1 becomes dominant under stress conditions. Future studies on the cooperation between different modes of lysosomal TSC complex tethering will reveal which mechanisms dominate upon different metabolic and stress stimuli.

Whereas G3BPs tether the TSC complex to lysosomes under nutrient sufficiency, oxidative (i.e., sodium arsenite) and heat stress induce the recruitment of TSC2 to G3BP1-positive SGs (Kosmas et al., 2021). SG recruitment of TSC2 is mediated by its interaction with high density lipoprotein binding protein (HDLBP, also named Vigilin), whose SG localization was discovered first in yeast (Wen et al., 2010). HDLBP appeared in two omics-wide analyses of SGs (Markmiller et al., 2018; Youn et al., 2018) and was shown recently to localize to SGs also in mammalian cells (Kosmas et al., 2021; Figure 1). Knockdown of HDLBP reduces TSC2 localization to SGs while not affecting SG formation, indicating that HDLBP mediates the SG recruitment of TSC2 (Kosmas et al., 2021). Interestingly, TSC2 deficiency enhances the number of G3BP1-positive SGs. In agreement, mTORC1 activity promotes SG assembly (Fournier et al., 2013; Sfakianos et al., 2018; Heberle et al., 2019), possibly constituting the mechanism *via* which TSC2 deficiency enhances SG assembly. It will be interesting to investigate whether stress-induced TSC2 translocation from the lysosomes to SGs elicits a positive feedback loop. By de-repression of mTORC1 at lysosomes, such positive feedback may enhance the formation of SGs and SG recruitment of TSC2. Intriguingly, under conditions of nutrient sufficiency (i.e., in the absence of SGs) not only the SG proteins G3BP1 and 2 (Prentzell et al., 2021), but also HDLBP (Wyant et al., 2018) reside at the lysosomes. HDLBP’s lysosomal function is still unknown and it remains open whether it also acts on lysosomal TSC2.

To conclude, G3BPs, SPAG5, and possibly HDLBP have dual roles at lysosomes and SGs (Thedieck et al., 2013; Kosmas et al., 2021; Prentzell et al., 2021). Of note, Liao et al. (2019) showed that ANXA11 (Annexin A11) tethers SGs to lysosomes for distal traveling in neurons. It is conceivable that such close proximity allows proteins to shuttle between lysosomes and SGs. Future research will tackle this question and may reveal the underlying mechanisms. The proximity of lysosomes and SGs may also explain observations that autophagy, one of the major functions of the lysosomal compartment, mediates SG clearance (Buchan et al., 2013; Marrone et al., 2018; Zhang et al., 2018; Silva et al., 2019) and their proper assembly (Seguin et al., 2014). mTORC1 is one of the key suppressors of autophagy as it inhibits autophagosome initiation by ULK1 and ATG13 (autophagy related 13) (Deleyto-Seldas and Efeyan, 2021). mTORC1 also inhibits TFEB and TFE3 (transcription factor EB, transcription factor binding to IGHM enhancer 3), major transcription factors of the autophagic-lysosomal pathway [reviewed by Noda et al. (2020)]. The involvement of SG proteins in lysosomal mTORC1 suppression may link them to autophagy and the turnover and assembly of the SG compartment.

### TSC Complex Tethers at Lysosomes and SGs in Human Disease

G3BP1 promotes proliferation of breast cancer cells (Winslow et al., 2013; Prentzell et al., 2021; Zhang et al., 2021) and in a TSC2-deficient tumor model (Kosmas et al., 2021). G3BP1 mRNA levels are increased in mouse and human TSC tumors [angiomyolipomas (AML), subependymal giant cell astrocytoma (SEGA), subependymal nodules (SEN)] (Kosmas et al., 2021) and in breast cancer (Winslow et al., 2013; Zhang et al., 2021). This suggests that G3BPs may be targets for tumor treatment. In keeping with this, inhibition of G3BP1 enhances apoptosis in TSC2-deficient cells *in vitro* (Kosmas et al., 2021). G3BP1 inhibition also prolongs tumor-free survival and represses tumor growth in a subcutaneous *in vivo* model derived from a TSC2-deficient renal tumor (Kosmas et al., 2021). This may have implications for the many proliferative lesions in TSC, which include renal AML, cardiac rhabdomyomas, and SEGAs (Henske et al., 2016). However, G3BP1 also suppresses migration in an mTORC1-dependent manner (Prentzell et al., 2021; Figure 1), suggesting that targeting G3BP1 may be contraindicated in some situations, such as breast cancer, in which lower levels of G3BP1, TSC1, and TSC2 are associated with reduced relapse-free survival (Prentzell et al., 2021). It is unknown whether G3BP1 controls TSC-associated tumors in human patients. G3BP1-dependent cell migration may be particularly important for women with LAM, the pulmonary manifestation of TSC, in which TSC2-deficient smooth muscle-like cells migrate to the lungs and cause emphysema-like lung destruction (Henske and McCormack, 2012). G3BP1-dependent migration may be also of clinical importance for cerebral cortical tubers in TSC, which are believed to arise from aberrant neuronal migration (Henske et al., 2016). The seemingly contradictory findings on G3BPs in tumors may result from G3BP1’s dual roles at SGs and lysosomes, respectively. Whereas SGs suppress cell death, making G3BP1 pro-tumorigenic, mTORC1 inhibition at lysosomes rather highlights the G3BPs as tumor suppressors. G3BPs also have a role in other oncogenic pathways, including RAS (Parker et al., 1996), NFKB1 (nuclear factor kappa B subunit 1) (Prigent et al., 2000), WNT (Bikkavilli and Malbon, 2011), and TGFB (Zhang et al., 2015). The G3BPs’ function that dominates in a given tumor may determine whether an intervention at the level of the G3BPs is pro- or anti-tumorigenic.

On a broader level, the new data on lysosomal and SG tethers of the TSC complex may impact our understanding of the pathogenesis and therapy of the many diseases in which dysregulation of the TSC complex-mTORC1 axis is observed. Diseases in which mTORC1 has a key role include the majority of human malignancies (Hoxhaj and Manning, 2020), as well as diabetes, obesity, and aging (Papadopoli et al., 2019; Liu and Sabatini, 2020). Understanding how the functions of G3BPs, PI(3,5)P2 and HDLBP in TSC subunit recruitment to lysosomes are integrated into the pathobiology of these diseases could have wide-ranging implications for human health. Like the G3BPs, also HDLBP (Yang et al., 2014) and PI(3,5)P2 (Hou et al., 2019; Ikonomov et al., 2019) control proliferation and migration of cancer cells. In agreement, altered HDLBP levels have been reported in different tumor entities (Yang et al., 2014; Woo et al., 2019), and PI(3,5)P2 and the G3BPs have been linked to malignancies and neuronal disorders (Wallroth and Haucke, 2018; Mandal, 2020; Prentzell et al., 2021). These disorders may arise, at least in part, from aberrant lysosomal TSC complex levels and mTORC1 activity.

## Outlook

To conclude, several mechanisms tether the TSC complex to lysosomes as well as to SGs, and control its inhibitory function toward mTORC1. Future research will unravel cooperation and competition between RHEB, RRAGs, G3BP1/2, PI(3,5)P2 and HDLBP in tethering the TSC complex to lysosomes and SGs and in controlling proliferation and migration under different metabolic and stress conditions. This may be clinically relevant for diseases characterized by dysregulated TSC complex and mTORC1 activity. G3BP proteins have been proposed as therapeutic targets based on their role in SG assembly (Zhang et al., 2012, 2019; Alam and Kennedy, 2019; Anisimov et al., 2019; Kosmas et al., 2021). However, their lysosomal TSC complex-tethering function warrants cautious evaluation of this concept in a tumor- and context-specific manner as G3BPs suppress oncogenic mTORC1 signaling. As HDLBP resides not only at SGs but also at lysosomes, it may give rise to pleiotropic effects similar to G3BPs that are to be investigated in future studies. G3BPs and HDLBP may represent indicators of mTORC1 activity with utility as predictive biomarkers for the response to drugs targeting mTORC1. Such applications will require careful investigation in clinical trials with inhibitors of mTORC1 and its upstream kinases.

## Author Contributions

UR and KT wrote the first draft of the manuscript. UR, MTP, AH, MCS, EH, CO, and KT contributed to the manuscript writing, read, and approved the final version. All authors contributed to the article and approved the submitted version.

## Conflict of Interest

The authors declare that the research was conducted in the absence of any commercial or financial relationships that could be construed as a potential conflict of interest.

## Publisher’s Note

All claims expressed in this article are solely those of the authors and do not necessarily represent those of their affiliated organizations, or those of the publisher, the editors and the reviewers. Any product that may be evaluated in this article, or claim that may be made by its manufacturer, is not guaranteed or endorsed by the publisher.
